# Preferred Islet Delivery Device Characteristics and Implantation Strategies of Patients With Type 1 Diabetes

**DOI:** 10.3389/ti.2023.11077

**Published:** 2023-10-16

**Authors:** Maarten C. Tol, Denise F. A. de Bont, Wouter P. C. Boon, Eelco J. P. de Koning, Aart A. van Apeldoorn

**Affiliations:** ^1^ Department of Internal Medicine, Leiden University Medical Center, Leiden, Netherlands; ^2^ LUMC Transplant Center, Leiden University Medical Center, Leiden, Netherlands; ^3^ Cell Biology-Inspired Tissue Engineering (cBITE), MERLN Institute for Technology-Inspired Regenerative Medicine, Maastricht University, Maastricht, Netherlands; ^4^ Copernicus Institute of Sustainable Development, Utrecht University, Utrecht, Netherlands; ^5^ Lighthouse Biomedical B.V., Maastricht, Netherlands

**Keywords:** islet transplantation, survey, type 1 diabetes, islet delivery device, patient preference

## Abstract

Islet delivery devices (IDDs) offer potential benefits for islet transplantation and stem cell-based replacement in type 1 diabetes. Little is known about patient preferences regarding islet delivery device characteristics and implantation strategies. Patient preferences for IDDs and implantation strategies remain understudied. We invited patients, parents and caregivers to fill in an online questionnaire regarding IDDs. An online survey gathered responses from 809 type 1 diabetes patients and 47 caregivers. We also assessed diabetes distress in a subgroup of 412 patients. A significant majority (97%) expressed willingness to receive an IDD. Preferred IDD attributes included a 3.5 cm diameter for 37.7% of respondents, while when provided with all options, 30.4% found dimensions unimportant. Respondents were open to approximately 4 implants, each with a 5 cm incision. Many favored a device functioning for 12 months (33.4%) or 24 months (24.8%). Younger participants (16–30) were more inclined to accept a 6 months functional duration (*p* < 0.001). Functional duration outweighed implant quantity and size (*p* < 0.001) in device importance. This emphasizes patients’ willingness to accommodate burdens related to IDD features and implantation methods, crucial for designing future beta cell replacement strategies.

## Graphical Abstract

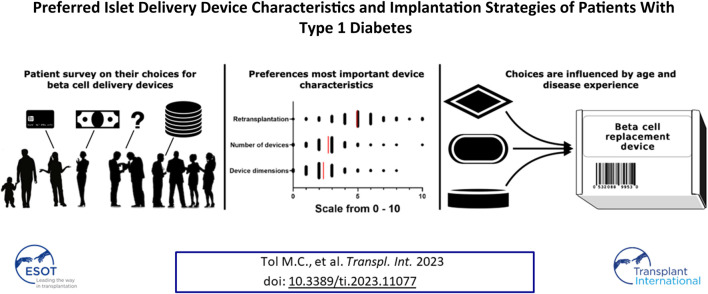

## Introduction

In type 1 diabetes (T1D) insulin-producing beta cells are destroyed by the immune system and patients are dependent on life-long administration of exogenous insulin for glycemic control and survival [1]. Allogeneic islet transplantation (ITx) in the portal vein of the liver is performed in a small group of patients with T1D and severe problems with glycemic control and/or complications [2]. Usually due to an insufficient transplantable islet mass, instant blood mediated inflammatory reaction (IBMIR) and long-term islet attrition in the liver only a minority of patients will have long-lasting clinically relevant islet graft function [3, 4]. In the last 2 decades, researchers have tried to improve the efficacy of ITx with so-called islet delivery devices (IDDs) [5–7].

Islet delivery devices exist in many sizes, shapes, with or without different compartments and are made from different (bio)materials [7–9]. It has been proposed that IDDs could support ITx at an extrahepatic site and potentially increase long-term functional capacity of transplanted islets [10]. There are two main types of islet delivery devices. Open devices support direct islet vascularization and efficient exchange of nutrients but require immunosuppressive medication [11]. Closed or immunoprotective devices are designed to prevent direct contact between the grafted cells and host immune cells thereby potentially preventing graft rejection. Recent developments in the generation of pluripotent stem cell-derived islets have focused more attention on the role of IDDs.

Despite the tremendous technical progress in the field of IDDs, there is a lack of information on user preferences. It is also unclear how diabetes distress and glycemic control affects preferences. In the current study, we evaluated preferences on IDD characteristics and implantation strategies in a cross-sectional study amongst a large group of Dutch patients with T1D.

## Material and Methods

### Subjects

Individuals aged 16 years and older with T1D were approached and invited to fill out a questionnaire about device preferences anonymously. One group of patients was approached by providing study information and a link to the questionnaire on various Dutch online platforms for patients with type 1 diabetes: Dutch Diabetes Research Foundation, Dutch Diabetes Association, Juvenile Diabetes Research Foundation (JDRF) Netherlands, Dutch Diabetes Meeting Point, diabetestype1.nl and Regenerative Medicine Crossing Borders (RegMedXB). Parents of children diagnosed with T1D younger than 16 years were also invited to participate. A second group of patients who had visited the diabetes outpatient clinic at the Leiden University Medical Center (LUMC) in the Netherlands during the past 2 years, were contacted by e-mail and invited to participate with a link to the online questionnaire.

### Questionnaires

We developed a web-based questionnaire (Qualtrics, Supplementary Appendix SA) for self-reported background information (age, sex, time since diagnosis T1D, most recent time in range (TIR), most recent hemoglobin A1c (HbA1c), current treatment and current treatment center) and preferences regarding specific aspects of islet delivery devices and implantation strategies. These preferences comprised implant sites and device characteristics such as the number of devices, the dimensions and the minimal duration of function. Respondents were invited to add explanatory remarks to their answers. Explanatory remarks were coded into categories and validated by a second investigator. The second group of patients from the LUMC were also requested to complete the 20-item Problem Areas In Diabetes (PAID) questionnaire [12]. The anonymous data were collected from October 2021 until May 2022.

### Data Handling and Analysis

Incomplete questionnaires were excluded from data analysis. A single respondent who indicated sex to be *other* was excluded from univariate and multivariate analysis by sex. Age categories were pre-specified in the questionnaire (16–30, 31–50, 51–70, >70 years, or parent/caregiver). PAID scores were categorized as low (0–16), moderate (17–39) or high (40–100) diabetes distress [13]. HbA1c levels were reported in mmol/mol Hb and if necessary converted from a percentage by the formula “mmol/mol Hb = (10.93 × %)–23.5”. Data were analyzed in RStudio (version 2023.03.1) and GraphPad Prism (version 9.3.1) with *α* = 0.05. Multivariate multinomial logistic regression was performed on variables with categorical outcomes, for all respondents with covariates age, sex, HbA1c and method of recruitment and just for the respondents of the diabetes outpatient clinic with age, sex, HbA1c and categorized PAID score. The most selected answers for the outcomes *preferred maximal size* and *minimal functional duration* were selected as reference. Comparison of continuous outcomes in multiple groups was done by repeated measures one-way ANOVA with Geisser-Greenhouse correction and Tukey’s multiple comparison test. Univariate analyses of binary outcome were performed with chi-square test.

## Results

### Respondent Characteristics

The online questionnaire was completed by 856 respondents (Supplementary Figure S1). The response rate was 43.1% amongst the approached patients of the LUMC diabetes outpatient clinic (412 respondents). Baseline characteristics of all respondents are presented in Table 1. The majority of respondents identified as female (58.1%) and were between the ages of 31 and 70 (71.8%). Forty-four percent of respondents had diabetes for more than 25 years. Mean self-reported HbA1c was 56.4 ± 12.4 mmol/mol Hb (*N* = 660) and mean self-reported TIR was 68.3% ± 17.3% (*N* = 465). The group of 412 patients also filled out the PAID questionnaire. The median PAID score was 25 (IQR: 12.5–39.1). Diabetes distress was determined to be low (PAID score 0–16) in 34%, moderate (PAID score 17–39) in 41% and high (PAID score 40–100) in 25% of respondents. Respondents recruited online were more often female, younger, had a shorter disease duration, and higher TIR than the patients contacted via the LUMC diabetes outpatient clinic (Supplementary Table S1).

**TABLE 1:** Patient characteristics.

	Overall (*N* = 856)
Sex	
Male	358 (41.8%)
Female	497 (58.1%)
Other	1 (0.1%)
Age (years)	
16–30	155 (18.1%)
31–50	311 (36.3%)
51–70	304 (35.5%)
>70	39 (4.6%)
Parent or caregiver	47 (5.5%)
Disease duration (years)	
<5	113 (13.2%)
5–15	196 (22.9%)
16–25	171 (20.0%)
>25	376 (43.9%)
Current treatment	
MDI^a^	403 (47.1%)
Pump therapy	440 (51.4%)
Other	13 (1.5%)
HbA1c, self-reported (mmol/mol Hb)	
Mean ± SD (N)	56.4 ± 12.4 (660)
Time in range, self-reported (%)	
Mean ± SD (N)	68.3 ± 17.3 (465)
PAID score	
Median (Q1–Q3, N)	25.0 (12.5–39.1, 412)
0–16	140 (34%)
17–39	169 (41%)
40–100	103 (25%)
Treatment center	
Local hospital	362 (42.3%)
University medical center	448 (52.3%)
Other	45 (5.3%)

^a^ MDI, multiple daily injections. PAID, problem areas in diabetes, indicating diabetes distress as low (0–16), moderate (17–39) or high (40–100). All units in N (%) unless otherwise indicated.

### Interest in Receiving an IDD

Nearly all (97%) respondents would like to receive an IDD (Table 2). Some respondents were willing to already take part in safety studies (44.0%), others would only accept the IDD after the completion of safety studies (44.4%).

**TABLE 2:** Patient preferences for receiving a device and the preferred implant strategy.

	Overall (*N* = 856)
Willingness to receive a device	
No	26 (3.0%)
Yes, as soon as possible (for example, by taking part in safety studies	377 (44.0%)
Yes, after completion of all safety studies	380 (44.4%)
Yes, after the device has been in the clinic for several years	73 (8.5%)
Preferred strategy	
An implant with average functioning cells, requiring 1 surgical procedure	52 (6.1%)
An implant with cells functioning well, requiring two surgical procedures	523 (61.1%)
An implant with excellent functioning cells, requiring 1 surgical procedure and 10 min of daily care to add oxygen	176 (20.6%)
No preference	105 (12.3%)

All units in N (%).

### Preferred Maximal Size

To explore the preferred maximal size of an implant, we surveyed 5 options (Table 3). We informed the respondents that a device would be flexible with a thickness of a credit card, and that it could be implanted, via a small incision, under the skin under local anesthesia at a location that would not be directly visible. Respondents were also informed that it could leave a scar. Most respondents (37.7%) preferred a maximal size corresponding to a FreeStyle Libre 2 sensor (diameter 3.5 cm), while 30.4% indicated that size was irrelevant. After correcting for sex, method of recruitment and HbA1c, respondents age >30 years compared to age 16–30 years were more likely to select a device with dimensions of a *2 Euro coin* (diameter 2.6 cm) rather than a *FreeStyle Libre 2 sensor* (*p*-values 0.007–0.048). Furthermore, parents/caregivers were less likely to opt for the choice “*size is irrelevant*” compared to the young reference group age 16–30 years (*p* = 0.043). Amongst the respondents from the outpatient clinic, having high diabetes distress increased the likelihood to select the option “*size is irrelevant*” over “*FreeStyle Libre 2 sensor*” compared to low diabetes distress (*p* = 0.003). Of the 602 respondents who left a comment at this question, 36.7% indicated that their choice for maximal size was motivated primarily by comfort: the device should not be visible nor hinder daily activities.

**TABLE 3:** Patient preferences for specific device characteristics and the expected improvements.

	Overall (*N* = 856)	Male (*N* = 358)	Female (*N* = 497)
Preferred maximal size			
2 Euro coin (diameter 2.5 cm)	95 (11.1%)	33 (9.2%)	62 (12.5%)
Freestyle Libre 2 sensor (diameter 3.5 cm)	323 (37.7%)	132 (36.9%)	191 (38.4%)
Credit card (8.5 cm × 5.5 cm)	166 (19.4%)	70 (19.6%)	95 (19.1%)
5 Euro banknote (12 cm × 6 cm)	12 (1.4%)	5 (1.4%)	7 (1.4%)
Size is irrelevant	260 (30.4%)	118 (33.0%)	142 (28.6%)
Maximal acceptable amount of implants			
Median (Q1–Q3)	4 (3–6.75)	4 (3–8)	4 (3–6)
Minimal expected functional duration			
3 months	113 (13.2%)	48 (13.4%)	65 (13.1%)
6 months	245 (28.6%)	108 (30.2%)	136 (27.4%)
12 months	286 (33.4%)	123 (34.4%)	163 (32.8%)
24 months	212 (24.8%)	79 (22.1%)	133 (26.8%)
Minimal expected improvement			
No more severe hyper- and hypoglycemia	104 (12.1%)	46 (12.8%)	58 (11.7%)
No more hyper- and hypoglycemia	301 (35.2%)	111 (31.0%)	190 (38.2%)
Less frequent insulin injections and monitoring	153 (17.9%)	73 (20.4%)	79 (15.9%)
Functional cure	298 (34.8%)	128 (35.8%)	170 (34.2%)

All units in N (%).

### Maximal Number of Implants

**FIGURE 1:**
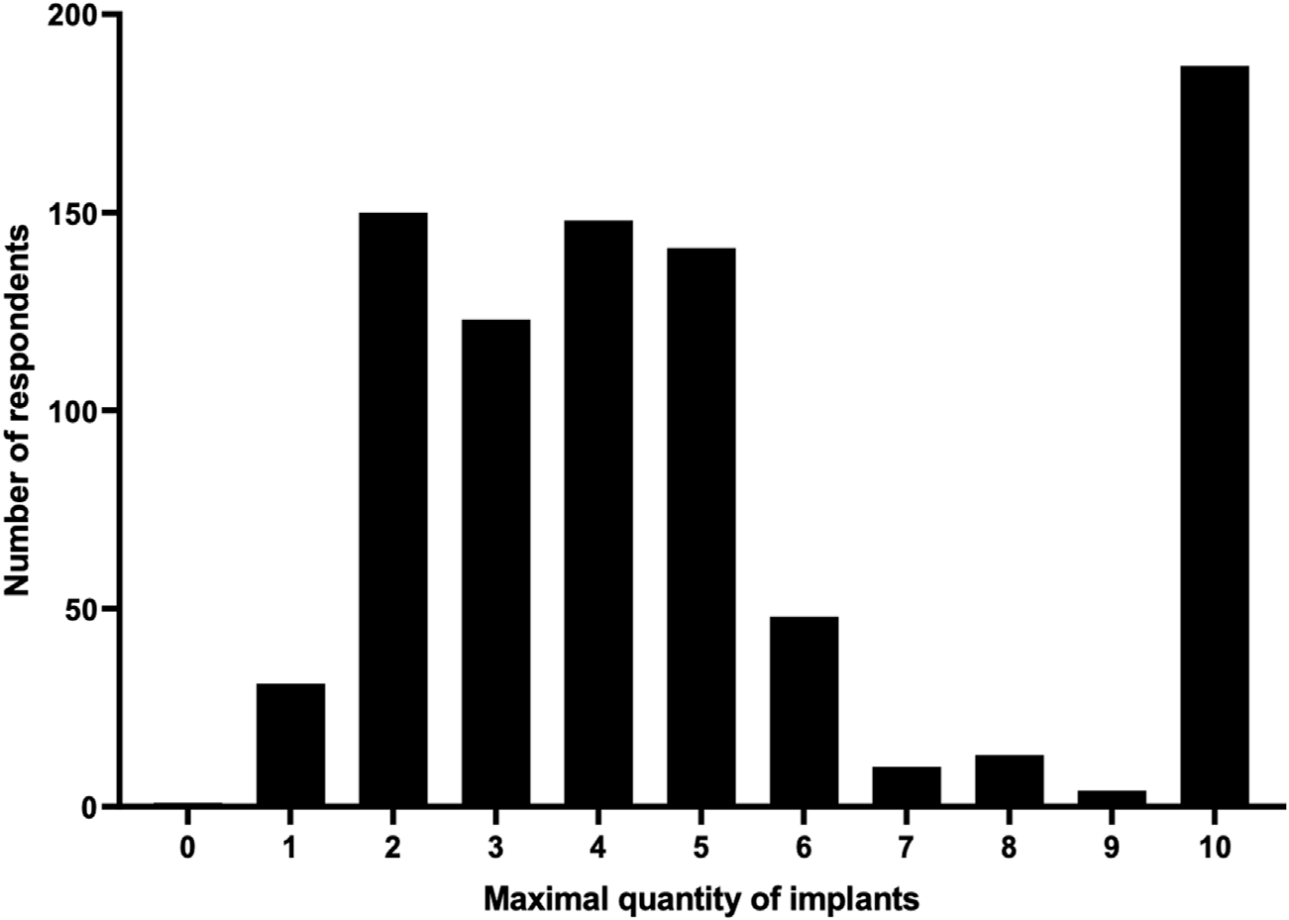
Maximal number of implants.

As it is likely that several implants would need to be implanted for maximal efficacy, we asked how many devices a respondent would simultaneously accept to be cured of type 1 diabetes given that an incision of 5 cm would be needed per implant. Respondents could choose between 0 and 10 devices. Respondents indicated a median of 4 (IQR 3–6.75) implants to be acceptable (Figure 1). The option for 10 implants was selected by 186 (21.7%) people, of whom 110 (59.1%) commented that the number of devices is not relevant if it ensures a cure. Of 479 respondents that left a comment, 10.9% indicated scar formation, 11.5% recovery after surgery and 22.3% a balance between cure and daily discomfort to be considerations in selecting a maximal number of devices.

### Minimal Expected Functional Duration

It is conceivable that the first generation of IDDs is functional for only a limited time. Therefore, respondents were informed that the cells in the implant would probably not be functional indefinitely, and that a replacement would be necessary under local anesthesia. We queried respondents what the minimal functional duration of an IDD should be before replacement (Table 3). A minimal functional duration of 3 months is the least desired option (13.2%). Replacement of an IDD of at most twice a year (minimal functional duration 6 months) was acceptable for 28.6%. A third (33.4%) of respondents would like the device to function for at least a year and 24.8% for at least 2 years. After correcting for method of recruitment, HbA1c and sex, respondents age 16–30 years were more likely to accept a minimal functional duration of 3–12 months when compared to those age 31–70 years (*p* < 0.001) and more likely to accept 6–12 months when compared to all other age categories (*p*-values 0.002–0.03).

Amongst respondents from the diabetes outpatient clinic, women were more likely than men to select a minimal functional duration of 24 months over 12 months (*p* = 0.01). Additionally, respondents age 31–50, and 51–70 years were more likely than those age 16–30 years to accept a minimal functional duration of 12 months compared to 6 months (*p* = 0.021 and *p* = 0.015, respectively). Of the 436 respondents who left a remark, 29.1% indicated that their choice revolved around minimizing emotional impact and impact on daily life due to hospital visits. Time to recovery and potential complications were important for 20%.

### Most Important Device Characteristic

**FIGURE 2:**
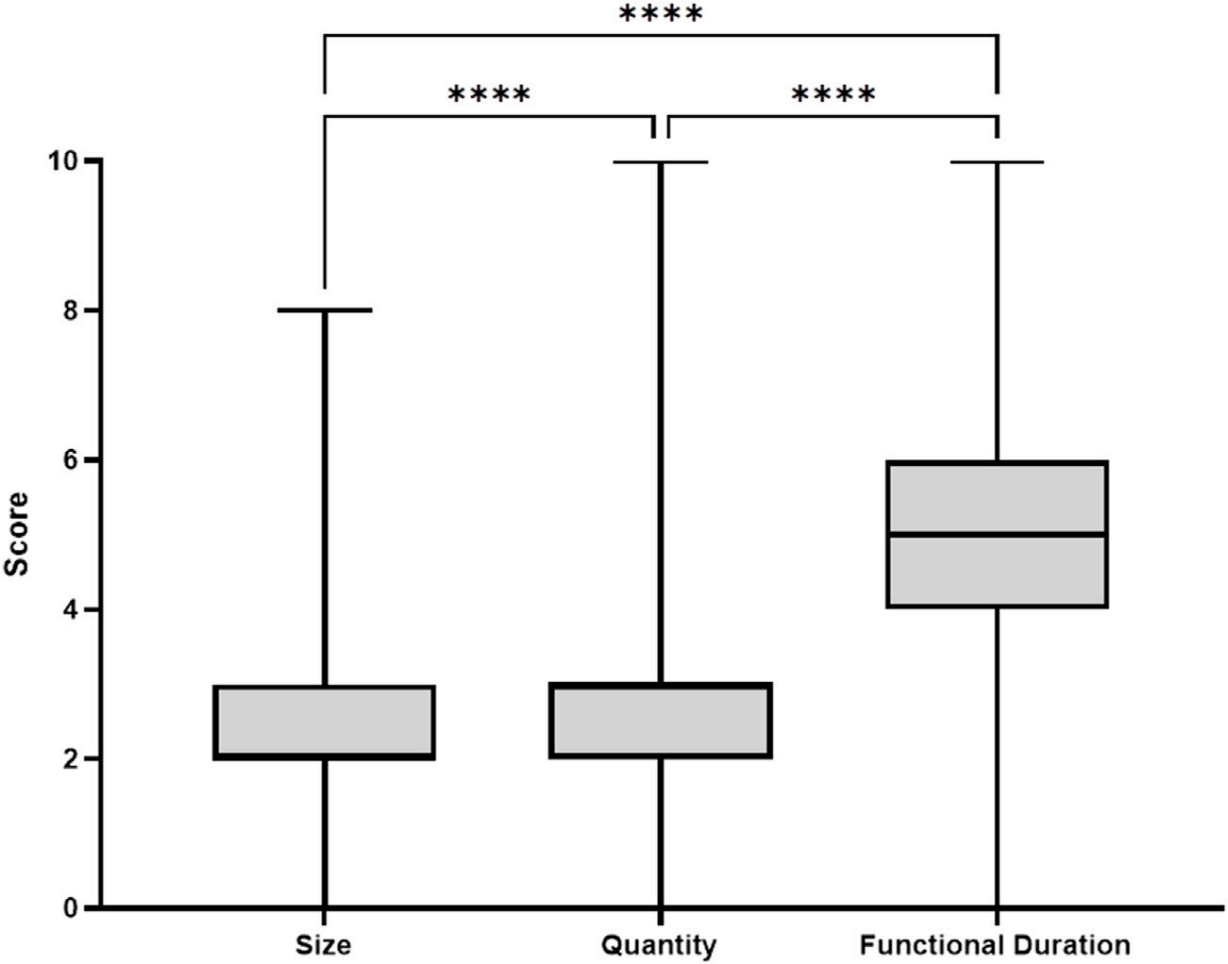
Scores indicating the most important device characteristic. Box indicated Q1–Q3.

To gain more insights into which device characteristic was considered most important, the respondents distributed 10 points between device characteristics *size*, *quantity* and *functional duration*. Respondents preferred *functional duration* over *quantity* and *size* (4.9 ± 1.8 vs. 2.7 ± 1.2 vs. 2.3 ± 1.3 points, respectively, *p* < 0.001, Figure 2).

### Respondents’ Preferences on Implantation of Islet Delivery Devices

To evaluate what implant sites were acceptable, we asked what body parts were most preferable. The three most acceptable sites were the abdomen (65.7%), upper leg (63.7%), and upper arm (54.8%), whereas the forearm (15.7%), chest (15.2%) and lower leg (14%) were the three least accepted implant sites (Table 4). The only difference in sex was that men were more positive about the chest as implant site than women (20.1% vs. 11.7%, *p* < 0.001).

**TABLE 4:** Patient preferences for specific implantation sites.

	Total (*N* = 856)	Male (*N* = 358)	Female (*N* = 497)	*p*-value
Abdomen				
Yes	562 (65.7%)	247 (69.0%)	314 (63.2%)	0.09
No	294 (34.3%)	111 (31.0%)	183 (36.8%)	
Upper leg				
Yes	545 (63.7%)	217 (60.6%)	327 (65.8%)	0.14
No	311 (36.3%)	141 (39.4%)	170 (34.2%)	
Upper arm				
Yes	469 (54.8%)	185 (51.7%)	283 (56.9%)	0.15
No	387 (45.2%)	173 (48.3%)	214 (43.1%)	
Butt				
Yes	362 (42.3%)	147 (41.1%)	215 (43.3%)	0.57
No	494 (57.7%)	211 (58.9%)	282 (56.7%)	
Hip				
Yes	321 (37.5%)	132 (36.9%)	189 (38.0%)	0.78
No	535 (62.5%)	226 (63.1%)	308 (62.0%)	
Back				
Yes	305 (35.6%)	115 (32.1%)	190 (38.2%)	0.077
No	551 (64.4%)	243 (67.9%)	307 (61.8%)	
Forearm				
Yes	134 (15.7%)	47 (13.1%)	87 (17.5%)	0.1
No	722 (84.3%)	311 (86.9%)	410 (82.5%)	
Chest				
Yes	130 (15.2%)	72 (20.1%)	58 (11.7%)	<0.001
No	726 (84.8%)	286 (79.9%)	439 (88.3%)	
Lower leg				
Yes	120 (14.0%)	55 (15.4%)	64 (12.9%)	0.35
No	736 (86.0%)	303 (84.6%)	433 (87.1%)	

The number and proportion of participants are shown for all variables.

### Minimal Expected Improvement

Islet delivery devices can potentially improve glycemic control and ideally lead to insulin independence. Respondents indicated in 34.8% of cases that they would only accept IDDs if it would cure them from diabetes (Table 3). In all other cases, various forms of improvement would also be acceptable. No longer suffering from hyperglycemia and hypoglycemia was the most selected non-curative improvement (35.2%).

### Preferred Device Strategy

Multiple device application strategies regarding islet delivery devices are currently considered. We surveyed the preference for three hypothetical scenarios (Table 2). Most respondents (61.1%) preferred a scenario with well-functioning cells that requires two surgical procedures over excellent-functioning cells requiring one surgical procedure and 10 min of daily care to supply oxygen (20.6%). A minority (6.1%) opted for moderately functioning cells after 1 surgical procedure. No preference was indicated by 12.3%.

## Discussion

The main outcome of our cross-sectional study in a large group of Dutch patients with type 1 diabetes is that patients with T1D are willing to accept a considerable burden of islet delivery device characteristics and implantation if this leads to a functional cure or clinically relevant improvement in hyper- and hypoglycemic events. Islet delivery devices could play an important future role in islet replacement strategies using insulin-producing cells from alternative cell sources such as pluripotent stem cells. Generating insights in the preferences of future recipients may support a smooth transition from IDD development to acceptance in the clinic.

In a previous report, Mohammadi et al. were the first to describe patient perspectives on implants for treatment of diabetes [14]. The results from their study indicated that patients with T1D prefer a device to be as small as possible and that a majority of the patients favored subcutaneous implantation. To gain more insight into what locations were preferred by patients we investigated which locations would be more preferable. Although not one location had a near total acceptance rate, most respondents accepted the abdomen, upper leg and upper arm which are sites that are often used for insulin injections and/or sensor placements.

The acceptance rate of IDDs was very high. Nearly all respondents indicated they would accept an IDD within the context of participation in a safety trial. T1D has a high disease burden [15] and diabetes distress partially mediates the relationship between depression and glycemic control [16]. It may not be surprising that device characteristics that may generate more discomfort are acceptable as long as it leads to a functional improvement or cure.

A morphomics framework was developed by McDermott et al. in which the body composition of 642 participants was evaluated using computed tomography images to analyze the maximal device dimensions [17]. In their model, maximal device dimensions were significantly larger in males, adults and dependent on BMI. The ideal device would be elliptical and could have an average surface area of 156 cm^2^ in males. This equals the size of two banknotes, which according to our study results is only acceptable by one-third of the respondents.

The limitations of our study were the use of a non-validated questionnaire to assess the preferences for device characteristics and the self-reported glycemic control. Self-report bias is an important limitation in studies using questionnaire. We accepted a putative difference between reported and actual HbA1c and TIR as these measures were used as an indicative marker rather than a prognostic or etiological factor. Selection bias may also have played a role as it is possible that non-interested patients with T1D did not start or complete the survey, and were therefore not considered or registered for data analysis. The response rate of 43.1% from the diabetes outpatient clinic similar to that of a different survey study amongst patients with diabetes [18]. The outcomes of our survey will allow researchers to incorporate device preferences of potential recipients at an early stage during device design and development [14].

## Conclusion

The vast majority of patients with type 1 diabetes would accept islet delivery devices when they become available. Respondents indicate that the minimal functional duration of an IDD is the most important characteristic. Implanting multiple IDDs is an acceptable strategy, although the potential discomfort while performing daily activities should be considered. The outcomes of this survey should not only serve as a recommendation for designing IDDs, but may also aid clinicians and researchers in setting up the appropriate clinical protocol for beta cell replacement strategies using cell delivery devices.

## Data Availability

The original contributions presented in the study are included in the article/Supplementary Material, further inquiries can be directed to the corresponding author.
