# Supplementary material for: Preferred Islet Delivery Device Characteristics and Implantation Strategies of Patients With Type 1 Diabetes
Source: Transpl Int. 2023 Oct 16;36:11077. doi: 10.3389/ti.2023.11077 (PMC10614671; doi:10.3389/ti.2023.11077)
Supplement: Supplementary file 1 [file DataSheet2.docx]

| Supplementary Table 1. Comparison in baseline characteristics between respondents recruited online versus diabetes outpatient clinic. | | | | | |  |
| --- | --- | --- | --- | --- | --- | --- |
|  | **Online (N=444)** | | **Diabetes outpatient clinic (N=412)** | **P-value** | |  |
| **Sex** |  | |  |  | |  |
| Male | 162 (36.5%) | | 196 (47.6%) | 0.0031 | |  |
| Female | 281 (63.3%) | | 216 (52.4%) |  | |  |
| Other | 1 (0.2%) | | 0 (0%) |  | |  |
| **Age (years)** |  | |  |  | |  |
| 16 - 30 | 99 (22.3%) | | 56 (13.6%) | <0.001 | |  |
| 31 - 50 | 162 (36.5%) | | 149 (36.2%) |  | |  |
| 51 - 70 | 131 (29.5%) | | 173 (42.0%) |  | |  |
| > 70 | 5 (1.1%) | | 34 (8.3%) |  | |  |
| Parent or caregiver | 47 (10.6%) | | 0 (0%) |  | |  |
| **Disease duration (years)** |  | |  |  | |  |
| < 5 | 93 (20.9%) | | 20 (4.9%) | <0.001 | |  |
| 5 - 15 | 112 (25.2%) | | 84 (20.4%) |  | |  |
| 16 - 25 | 86 (19.4%) | | 85 (20.6%) |  | |  |
| > 25 | 153 (34.5%) | | 223 (54.1%) |  | |  |
| **Current treatment** |  | |  |  | |  |
| MDI* | 185 (41.7%) | | 218 (52.9%) | 0.0042 | |  |
| Pump therapy | 251 (56.5%) | | 189 (45.9%) |  | |  |
| Including (is)CGM* | |  | |  |  | |
| AID* | | 12 | | 12 |  | |
| No information on AID | | 23 | | 8 |  | |
| No information on (is)CGM) | | 216 | | 169 |  | |
| Other | 8 (1.8%) | | 5 (1.2%) |  | |  |
| **HbA1c (mmol/mol Hb)** |  | |  |  | |  |
| Mean ± SD (N) | 56.1 ± 12.5 (365) | | 56.8 ± 12.2 (295) | 0.51 | |  |
| **Time in range (%)** |  | |  |  | |  |
| Mean ± SD (N) | 70.4 ± 17.1 (259) | | 65.7 ± 17.2 (206) | 0.004 | |  |
| **Treatment center** |  | |  |  | |  |
| Local hospital | 342 (77.0%) | | 20 (4.9%) | <0.001 | |  |
| University medical center | 60 (13.5%) | | 388 (94.2%) |  | |  |
| Other | 41 (9.2%) | | 4 (1.0%) |  | |  |
| *MDI: Multiple daily injections. (is)CGM: (intermittent scanning) continuous glucose monitoring. AID: automatic insulin delivery. All units in N (%) unless otherwise indicated. | | | | | |  |

| Supplementary Table 2. Comparison in outcome parameters between respondents recruited online versus diabetes outpatient clinic. | | | |
| --- | --- | --- | --- |
| All units in N (%) unless otherwise indicated | **Online (N=444)** | **Diabetes outpatient clinic (N=412)** | **P-value** |
| **Maximal preferred size** |  |  |  |
| 2 Euro coin (diameter 2.5cm) | 51 (11.5%) | 44 (10.7%) | 0.86 |
| Freestyle Libre 2 sensor (diameter 3.5cm) | 173 (39.0%) | 150 (36.4%) |  |
| Credit card (8.5 x 5.5cm) | 84 (18.9%) | 82 (19.9%) |  |
| 5 Euro banknote (12 x 6cm) | 5 (1.1%) | 7 (1.7%) |  |
| Size is irrelevant | 131 (29.5%) | 129 (31.3%) |  |
| **Minimal expected functional duration** |  |  |  |
| 3 months | 63 (14.2%) | 50 (12.1%) | 0.49 |
| 6 months | 125 (28.2%) | 120 (29.1%) |  |
| 12 months | 140 (31.5%) | 146 (35.4%) |  |
| 24 months | 116 (26.1%) | 96 (23.3%) |  |
| **Minimal expected improvement** |  |  |  |
| No more severe hyper- and hypoglycemia | 56 (12.6%) | 48 (11.7%) | 0.74 |
| No more hyper- and hypoglycemia | 161 (36.3%) | 140 (34.0%) |  |
| Less frequent insulin injections and monitoring | 80 (18.0%) | 73 (17.7%) |  |
| Functional cure | 147 (33.1%) | 151 (36.7%) |  |
| **Maximal acceptable number of implants** |  |  |  |
| Median (Q1 – Q3) | 4 (3 – 7) | 4 (3 – 6) | 0.58 |
| **Preferred strategy** |  |  |  |
| An implant with average functioning cells, requiring 1 surgical procedure | 27 (6.1%) | 25 (6.1%) | 0.98 |
| An implant with cells functioning well, requiring 2 surgical procedures | 269 (60.6%) | 254 (61.7%) |  |
| An implant with excellent functioning cells, requiring 1 surgical procedure and 10 minutes of daily care to add oxygen | 94 (21.2%) | 82 (19.9%) |  |
| No preference | 54 (12.2%) | 51 (12.4%) |  |
| **Willingness to receive a device** |  |  |  |
| No | 14 (3.2%) | 12 (2.9%) | 0.94 |
| Yes, as soon as possible (for example by taking part in safety studies | 196 (44.1%) | 181 (43.9%) |  |
| Yes, after completion of all safety studies | 194 (43.7%) | 186 (45.1%) |  |
| Yes, after the device has been in the clinic for several years | 40 (9.0%) | 33 (8.0%) |  |

| Supplementary Table 3. Outcome parameters by age. | | | | | |
| --- | --- | --- | --- | --- | --- |
|  | **16 - 30 (N=155)** | **31 - 50 (N=311)** | **51 - 70 (N=304)** | **> 70 (N=39)** | **Parent or caregiver (N=47)** |
| **Maximal preferred size** |  |  |  |  |  |
| 2 Euro coin  (Diameter 2.5cm) | 9 (5.8%) | 41 (13.2%) | 32 (10.5%) | 6 (15.4%) | 7 (14.9%) |
| Freestyle Libre 2 sensor (diameter 3.5cm) | 58 (37.4%) | 125 (40.2%) | 102 (33.6%) | 14 (35.9%) | 24 (51.1%) |
| Credit card (8.5 x 5.5cm) | 29 (18.7%) | 62 (19.9%) | 63 (20.7%) | 6 (15.4%) | 6 (12.8%) |
| 5 Euro banknote (12 x 6cm) | 4 (2.6%) | 4 (1.3%) | 2 (0.7%) | 1 (2.6%) | 1 (2.1%) |
| Size is irrelevant | 55 (35.5%) | 79 (25.4%) | 105 (34.5%) | 12 (30.8%) | 9 (19.1%) |
| **Minimal expected functional duration** |  |  |  |  |  |
| 3 months | 41 (26.5%) | 31 (10.0%) | 37 (12.2%) | 2 (5.1%) | 2 (4.3%) |
| 6 months | 55 (35.5%) | 86 (27.7%) | 81 (26.6%) | 12 (30.8%) | 11 (23.4%) |
| 12 months | 39 (25.2%) | 110 (35.4%) | 105 (34.5%) | 15 (38.5%) | 17 (36.2%) |
| 24 months | 20 (12.9%) | 84 (27.0%) | 81 (26.6%) | 10 (25.6%) | 17 (36.2%) |
| **Minimal expected improvement** |  |  |  |  |  |
| No more severe hyper- and hypoglycemia | 21 (13.5%) | 44 (14.1%) | 29 (9.5%) | 5 (12.8%) | 5 (10.6%) |
| No more hyper- and hypoglycemia | 61 (39.4%) | 109 (35.0%) | 105 (34.5%) | 12 (30.8%) | 14 (29.8%) |
| Less frequent insulin injections and monitoring | 36 (23.2%) | 50 (16.1%) | 52 (17.1%) | 7 (17.9%) | 8 (17.0%) |
| Functional cure | 37 (23.9%) | 108 (34.7%) | 118 (38.8%) | 15 (38.5%) | 20 (42.6%) |
| **Maximal acceptable number of implants** |  |  |  |  |  |
| Median (Q1 - Q3) | 5 (3 - 10) | 4 (3 - 7) | 5 (3 - 6) | 4 (2.5 - 5) | 4 (3 - 5) |
| **Willingness to receive a device** |  |  |  |  |  |
| No | 1 (0.6%) | 7 (2.3%) | 11 (3.6%) | 2 (5.1%) | 5 (10.6%) |
| Yes, as soon as possible (for example by taking part in safety studies | 77 (49.7%) | 134 (43.1%) | 140 (46.1%) | 15 (38.5%) | 11 (23.4%) |
| Yes, after completion of all safety studies | 63 (40.6%) | 142 (45.7%) | 136 (44.7%) | 18 (46.2%) | 21 (44.7%) |
| Yes, after the device has been in the clinic for several years | 14 (9.0%) | 28 (9.0%) | 17 (5.6%) | 4 (10.3%) | 10 (21.3%) |
| **Preferred strategy** |  |  |  |  |  |
|  |  |  |  |  |  |
| An implant with average functioning cells, requiring 1 surgical procedure | 6 (3.9%) | 22 (7.1%) | 17 (5.6%) | 5 (12.8%) | 2 (4.3%) |
| An implant with cells functioning well, requiring 2 surgical procedures | 104 (67.1%) | 182 (58.5%) | 187 (61.5%) | 22 (56.4%) | 28 (59.6%) |
| An implant with excellent functioning cells, requiring 1 surgical procedure and 10 minutes of daily care to add oxygen | 29 (18.7%) | 71 (22.8%) | 60 (19.7%) | 6 (15.4%) | 10 (21.3%) |
| No preference | 16 (10.3%) | 36 (11.6%) | 40 (13.2%) | 6 (15.4%) | 7 (14.9%) |
| All units in N (%) unless otherwise indicated. | | | | | |

Did not meet age criterium (n=6)

Patients surveyed from the outpatient clinic (n=978)

Started the survey (n=483)

Assessed for eligibility (n=422)

Included in analysis (n=412)

Patients surveyed online

Completed surveys

(n=450)

Assessed for eligibility (n=450)

Included in analysis (n=444)

Did not complete survey (n=61)

Did not respond (n=495)

No informed consent (n=4)

No type 1 diabetes (n=6)

Total number of included respondents (n=856)

Supplementary Figure 1: flowchart of inclusions.
