# Supplementary material for: Preferred Islet Delivery Device Characteristics and Implantation Strategies of Patients With Type 1 Diabetes
Source: Transpl Int. 2023 Oct 16;36:11077. doi: 10.3389/ti.2023.11077 (PMC10614671; doi:10.3389/ti.2023.11077)
Supplement: Supplementary file 2 [file DataSheet1.docx]

**Appendix A**

**Questionnaire presented to patients, caretakers, and parents of children with type 1 diabetes**

The University of Maastricht (the Netherlands) is working on implants to treat patients with type 1 diabetes. These implants can return part of the insulin secretion. Through this questionnaire, we would like to find out what your wishes and expectations are as a possible target group. You can fill in this questionnaire if you or your child has been diagnosed with type 1 diabetes.

Current treatment to become insulin-independent:

- Islet transplantation: here the islets from a donor are transplanted unprotected into the body. There is a chance that the islets die prematurely.

New treatment to become insulin-independent:

- Transplantation of an implant containing insulin-producing cells. The implant prevents the cells from dying.

It takes about 5 to 10 minutes to fill in this questionnaire, this can be done on a phone, tablet, or computer. First, we ask a number of questions about the implant, followed by a number of background questions. The answers are processed anonymously.

Some questions have additional information, you can recognize this by words that are underlined. Unfortunately, this does not work on a phone.

The implant is expected to be flexible with a thickness similar to that of a bank card. The implant is placed through a small incision in the skin with local anesthetic, in a location that is not immediately visible. This operation can be performed as a day treatment. This can leave you with a scar.

**What is the maximum size of the implant that is still reasonable for you?**

Note: The thickness and flexibility is the same for each of the options.

- 2 euro coin (25 mm)
- Free style libre sensor (35 mm)
- Debit card (85 x 55 mm)
- 5 euro note (120 x 60 mm)
- Dimensions are not relevant for me

**What are important considerations for you in the answer you entered?**

Note: You are not required to enter anything here.

The size of the implant depends on the number of cells that need to be transplanted. The transplant can become more effective when more cells are transplanted. As a result, multiple implants have to be placed.

Here you should assume that a **5 cm incision is made for each implant**.

|  | 1 | 2 | 3 | 4 | 5 | 6 | 7 | 8 | 9 | 10 |
| --- | --- | --- | --- | --- | --- | --- | --- | --- | --- | --- |

| **How many implants (and therefore cuts) would you like to receive as a maximum at the same time for a cure for type 1 diabetes?** | 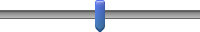 |
| --- | --- |

**What are important considerations for you in the answer you entered?**

Note: You are not required to enter anything here.

_______________________________________________________________

The ultimate goal is to develop an implant that will last as long as possible. Unfortunately, the cells in the implant will not live forever. The implant will have to be replaced occasionally with a new implant. The implant can be easily replaced with local anesthetic.

**How long should the implant produce insulin minimally before you need to be transplanted again?**

- Every 3 months (4x per year)
- Every 6 months (2x per year)
- Every 12 months (1x per year)
- Every 24 months (1x per 2 years)

**What are important considerations for you in the answer you entered?**

Note: You are not required to enter anything here.

________________________________________________________________

You just received a number of questions about the dimensions of the implant, the number of implants, and the duration of function.

**Which of these three properties do you consider most important?**

Divide 10 points between the three options, more points is more important.

Dimensions of the implant : _______

Number of implants : _______

Duration of function : _______

Total : ________

**Where would you like to receive the implant?**

One or multiple answers are possible.

- Upper arm
- Upper leg
- Abdomen
- Forearm
- Lower leg
- Chest
- Back
- Hips
- Butt

The cells in the implant will produce insulin, making you less dependent on the administration of insulin. This can improve the quality of your life.

**What is the minimal improvement you would like to see after transplantation?**

- No more severe hypers and hypos
- No more hypers and hypos
- Less (frequent) insulin injections / glucose monitoring
- Cure - no insulin injections necessary

Preparations are currently underway to obtain approval to test the implant in humans. A number of safety studies then will take place to show that the implant is safe. Only then may the implant be used on a large scale in the clinic.

Note: With this questionnaire, we try to gain insight into your preferences; we do not recruit patients to participate in safety studies through this questionnaire.

**Would you be interested in receiving this implant?**

- No
- Yes, as soon as possible (for example by participating in a safety study)
- Yes, but only after all safety studies have been completed
- Yes, but only after the implant has been in use for several years in the clinic

Currently, various researchers and companies worldwide are working on various implants.

**Which of the following hypothetical example implants do you prefer?**

- An implant with moderate effect for which you need **1 operation**.
- An implant with good effect for which you need **2 operations**.
- An implant with excellent effect for which you need **1 operation and administer oxygen daily. This takes 10 minutes daily.**
- No idea/no preference.

Finally, we ask you a few questions about your (diabetes) background.

**What is your age?**

- 16 – 30
- 31 – 50
- 51 – 70
- > 70
- Parent/caregiver of a child

**What is your gender?**

If you are a parent/caregiver, you can enter the gender of your child.

- Male
- Female
- Other

**What is your treatment center?**

- Local hospital
- University hospital
- Diabetes treatment Center

**How long have you or your child been diagnosed with type 1 diabetes?**

- < 5 years
- 5 – 15 years
- 16 – 25 years
- > 25 years

**What is your (or your child's) current treatment?**

- Insulin pen
- Insulin pump. If so, what system are you using (e.g.: Omnipod, MiniMed™ 780G)?

--------------------------------

- Other

**What is your (or your child's) last measured HbA1c value?** If unknown, please write "n/a".

________________________________________________________________

**What is your (or your child's) time in range?** If unknown, please write "n/a".

________________________________________________________________

On a scale of 1 to 10, what is your (or your child’s) burden from glucose fluctuations and complications?
Note: 1 = no burden / no influence                           10 = very burdened / a lot of influence

|  | 1 | 2 | 3 | 4 | 5 | 6 | 7 | 8 | 9 | 10 |
| --- | --- | --- | --- | --- | --- | --- | --- | --- | --- | --- |

| **What is your (or your child’s) burden from glucose fluctuations?** | 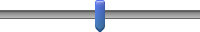 |
| --- | --- |
| **What is your (or your child’s) burden from diabetic complications?** | 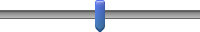 |
| **How much does diabetes influence your (or your child’s) daily life?** | 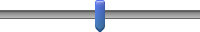 |
